# The clinical significance of *c-Kit* mutations in metastatic oral mucosal melanoma in China

**DOI:** 10.18632/oncotarget.19746

**Published:** 2017-07-31

**Authors:** Xuhui Ma, Yunteng Wu, Tian Zhang, Hao Song, Houyu Jv, Wei Guo, Guoxin Ren

**Affiliations:** ^1^ Department of Oral and Maxillofacial-Head and Neck Oncology, Shanghai Ninth People’s Hospital, School of Medicine, Shanghai Jiaotong University, Shanghai 200011, China

**Keywords:** oral mucosal melanomas, distal metastasis, c-Kit, c-Kit mutation, imatinib

## Abstract

*c-Kit* mutations are frequently detected in mucosal melanomas, but their clinical significance in metastatic oral mucosal melanomas (OMM) remains unclear. The main purpose of this study was to investigate the clinical and pathological features of metastatic OMMs with *c-Kit* mutations and the efficiency of the tyrosine kinase inhibitor imatinib in treating metastatic OMMs. We found thatresidual primary lesion and neck lymph nodes could act as independent prognostic factors in metastatic OMM patients. *c-Kit* mutations were detected in 22 out of 139 (15.8%) metastatic OMM patients. Under chemotherapy, the overall survival (OS) of *c-Kit* mutant patients was significantly shorter than that of wild-type patients. The Ki67 expression was significantly higher in *c-Kit* mutant patients than in wild-type patients. In distant metastatic OMM patients with *c-Kit* mutations, the treatment with c-Kit inhibitor resulted in a better OS. In conclusion, residual primary lesion, cervical lymph nodes and *c-Kit* mutations act as adverse prognostic factors of metastatic OMMs. The Kit inhibitor imatinib could benefit metastatic OMM patients with *c-Kit* mutations.

## INTRODUCTION

Melanoma is a highly aggressive malignant tumor with biologically distinct subtypes [1, 2]. Oral mucosal melanoma (OMM) is an exceedingly rare neoplasm [3–5], accounting for less than 1% of all melanomas in the USA and about 7.5% in Asians [6, 7]. The dismal prognosis of melanoma results mainly from distant metastasis, and the median overall survival (OS) is less than 10 months in advanced patients [8–10]. More ominously, some traditional cytotoxic chemotherapeutic agents, including dacarbazine and temozolomide, have been reported to be ineffective against metastatic OMMs [11–14]. Clearly, there is a need to find a more effective treatment for metastatic OMMs.

Some genetic mutations, such as active mutations of *Braf* and *c-Kit*, may act as molecular hubs promoting the development of melanomas and thus can be potential therapeutic targets [15]. Small molecule inhibitors of both Braf (Dabrafenib) and c-Kit (Imatinib) have shown promising results for advanced cutaneous melanoma patients. In comparison with cutaneous melanoma that has a high rate of *Braf* mutations, OMM rarely harbors *Braf* mutations [16–18], which hampered the use of Braf inhibitors in OMM patients. In addition, there is increasing evidence that OMM harbors *c-Kit* mutations [19–21], indicating that imatinib, a c-Kit inhibitor, may be beneficial for advanced OMM patients.

The *c-Kit* gene encodes CD117, a type III transmembrane receptor tyrosine [22, 23]. *c-Kit* protein (Kit) includes five distinct domains: a glycosylated extracellular ligand binding domain (coded by exons 1-9), a hydrophobic transmembrane domain (coded by exon 10), an intracellular juxtamembrane domain (coded by exon 11), and two tyrosine kinase domains (coded by exons 12-21) [24]. The intracellular juxtamembrane domain has been shown to be autoinhibitory, which can prevent Kit activation in the absence of extracellular ligand. As the ligand such as stem cell factor binds to the extracellular domain, the Kit receptors dimerize to each other, resulting in autophosphorylation of the tyrosine kinase domains and activation [23]. Once activated, Kit can initiate the activation of a variety of downstream pathways including the MAPK/MEK and PI3K/AKT pathways which play important roles in cancer development [23, 25].

Activating *c-Kit* mutations in the juxtamembrane and other domains has been considered as an oncogene and also a therapeutic target in various tumors [23]. *c-Kit* mutation has been detected in various mucosal melanomas [19, 20], but it remains unclear whether it is a prognostic factor or therapeutic target in distant metastatic OMMs. The main purpose of this study was to investigate the clinical and pathological features of metastatic OMMs with *c-Kit* mutations and the efficiency of the tyrosine kinase inhibitor imatinib in treating metastatic OMMs. In doing so, we investigated the clinical manifestations, histopathology, treatment, and outcomes of metastatic OMM patients, aiming to explore the prognostic factors of metastatic OMMs and the efficacy of imatinib on metastatic OMMs with *c-Kit* mutations.

## RESULTS

### Patient characteristics

A total of 139 patients with distant metastases were enrolled in this study, including 75 males (53.96%) and 64 females (46.04%). The median age of these patients at first metastasis was 61 years (19–75 years). The primary lesion was located in the hard palate in 85 patients (61.15%; Supplementary Figure 1A), maxillary gum in 28 patients (20.14%; Supplementary Figure 1B), mandible gum in 17 patients (12.23%; Supplementary Figure 1C), and other sites such as buccal and tongue in 9 patients (6.47%; Supplementary Figure 1D), respectively. At the diagnosis of distant metastasis, 103 patients had metastases at only one site, and the rest 36 patients had metastases in two or more organs. Residual or recurrent lesions were detected in the oral cavity of 23 patients, while positive neck nodes were detected in 25 patients (Table 1). Of these 139 patients, 22 (15.8%) harbored *c-Kit* mutations, including 9 (40.9%) with mutations in exon 11, 5 (22.7%) in exon 13, 3 (13.6%) in exon 18, 2 in exon 17 (9.1%) and 9 (9.1%), and 1 (4.5%) in both exon 13 and exon 9, respectively (Table 2).

**Table 1:** OS of metastatic OMMs patients by prognostic variables

Variable	NO.	MedinaOS (weeks)	*P*	
			Unviariate	Multivariate
Sex				
Male	75	32	0.665	-
Female	64	32		
Age, years				
< 60	61	32	0.708	-
≥ 60	78	33		
Primary site				
Palate	85	32	0.857	-
Maxillary gum	28	34		
Mandible gum	17	33		
Other site	9	35		
ECOG PS score				
0	47	31	0.206	-
1	92	34		
Primary lesion				
Positive	23	26	<0.001	<0.001
Negative	116	35		
CLN				
Positive	25	27	<0.001	<0.001
Negative	114	35		
*c-Kit* mutation				
Positive (Group 2+3)	22	32	0.583	-
Negative (Group 1)	117	32		
Site of metastases				
Single site	103	33	0.001	0.077
Multiple sites	36	31		

**Table 2:** Specific *c-Kit* mutations in each exon

*c-Kit* mutation	*c-Kit* mutant with chemo (n=10)			*c-Kit* mutant with imatinib (n=12)			Total (n=22)
	No. (%)	mutation	No.	No. (%)	mutation	No.	No. (%)
Exon 9	1 (10)			1 (8.3)			2 (9.1)
		F504L	1		F504L	1	
Exon 11	4 (40)			5 (41.7)			9 (40.9)
		L576P	3		L576P	4	
		V559A	1		V559A	0	
		-	-		V560D	1	
Exon 13	2 (20)			3 (25)			5 (22.7)
		K642E	2		K642E	3	
Exon 17	2 (20)			0 (0)	-	0	2 (9.1)
		D816H	1				
		N822I	1				
Exon 18	1 (10)			2 (16.7)			3 (13.6)
		A829P	1		A829P	2	
Multiple mutations	0 (0)			1 (8.3)			1 (4.5)
		-	-		F504L(Exon 9)+K642E(Exon13)	1	

### Primary lesion and positive cervical lymph node (CLN) were significant prognostic factors for distant metastatic OMMs

**Figure 1:**
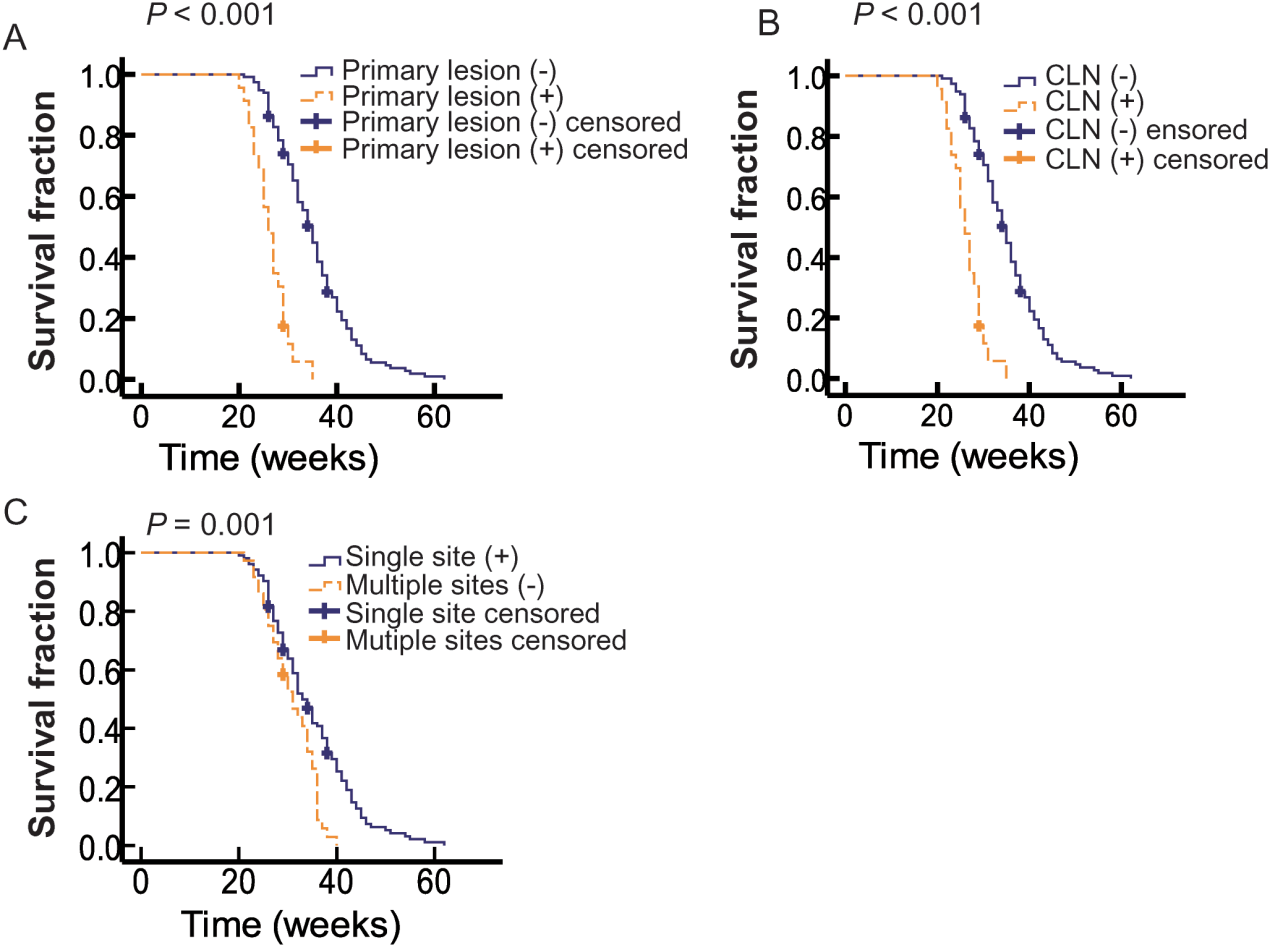
The correlation of OS with different clinical variables **(A)** Kaplan-Meier curves showed the OS by residual primary lesion. Solid blue line indicates patients without primary lesion (n=116) and the dotted yellow line represents patients harbor primary lesion (n=23). **(B)** Correlation of OS and cervical lymph node (CLN). Solid blue line indicates patients without CLN (n=114) and the dotted yellow line represents patients with CLNs (n=25). **(C)** Kaplan-Meier curves showed the OS by site of metastases. Solid blue line indicates patients with single site of metastases (n=103) and the dotted yellow line represents patients with multiple sites of metastases (n=36).

Figure 1A showed that patients with residual primary lesion had worse outcomes than those without residual primary lesion, with a median OS of 26 and 35 weeks, respectively (*P* < 0.001). Figure 1B showed that patients with positive CLNs had worse outcomes than those with negative CLNs, with a median OS of 27 and 35 weeks, respectively (*P* < 0.001). Multivariate analysis suggested that both primary lesion and positive neck nodes could act as an independent prognostic factor for distant metastatic OMMs (Table 1).

### Multiple distant lesions resulted in worse outcome than a single distant lesion

Nearly 3/4 patients had only one distant lesion, and, as a consequence, they had a longer median OS (33 weeks) than patients with multiple distant lesions (31 weeks) (*P* = 0.001; Figure 1C). However, this variable was not an independent prognostic factor in the multivariate analysis (Table 1).

### *c*-*Kit* mutation was an adverse prognostic factor for distant metastatic OMMs

**Figure 2:**
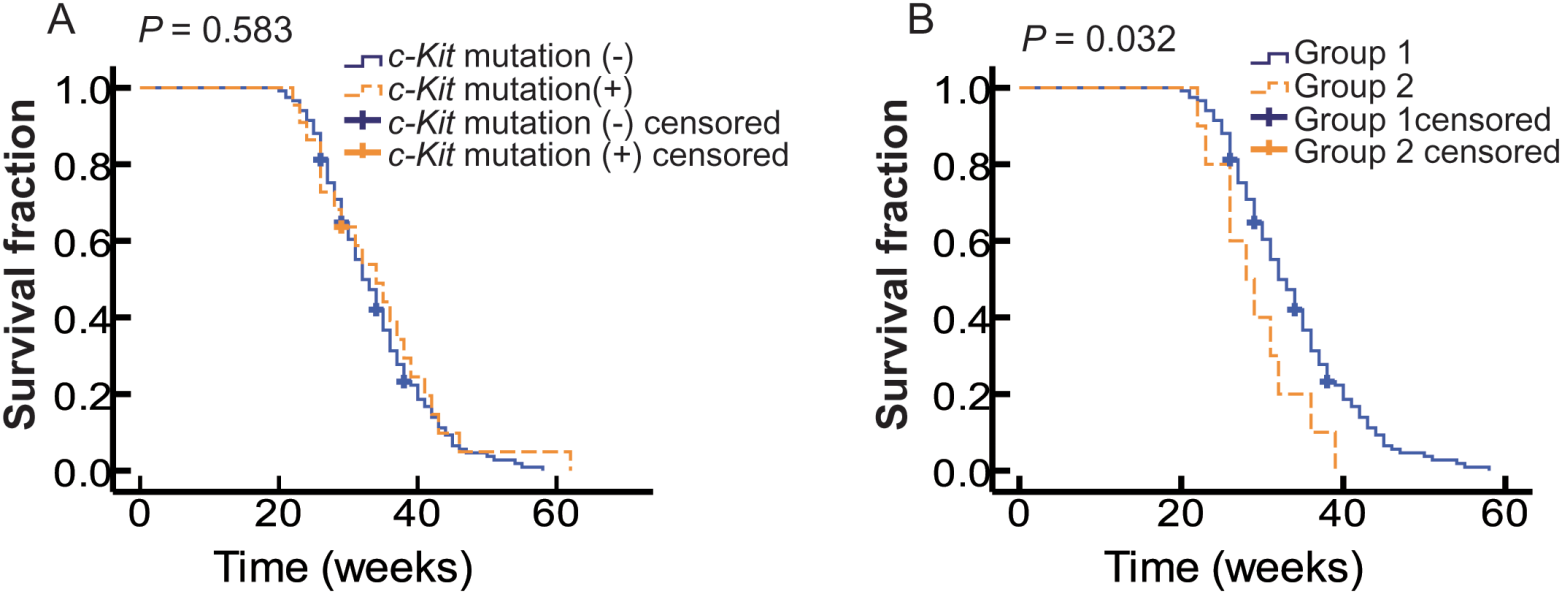
The correlation of OS with *c-Kit* mutations **(A)** Kaplan-Meier curves of OS of patients with WT *c-Kit* gene (solid blue line, n=117) and mutant *c-Kit* gene (dotted yellow line, n=22). **(B)** Kaplan-Meier curves of OS of patients received chemotherapy. Solid blue line indicates patients with WT *c-Kit* gene (Group1, n=117) and the dotted yellow line mutant *c-Kit* gene (Group2, n=10).

It has been recently reported that *c-Kit* mutation may be a negative prognostic factor in melanoma [26], but it remains unknown whether it could affect the OS of metastatic OMMs patients. In this study, we found no significant difference in OS between WT and *c-Kit* mutant patients (Table 1 and Figure 2A). However, as such findings could be confounded by different treatment strategies received by *c-Kit* mutant patients, we divided patients into 3 subgroups depending on their status of *c-Kit* mutation and treatment: wild-type patients (Group 1, *n* = 117), patients with *c-Kit* mutations and chemotherapy (Group 2, *n* = 10), and patients with *c-Kit* mutations and imatinib treatment (Group 3, *n* = 12). There was no significant difference in patient characteristics, including CLN, the status of primary lesion, and the sites of distant metastases, among the three groups (Table 3). However, we found that the median OS of Group 2 was significantly shorter than that of Group 1 (28 VS 32 weeks, *P* = 0.032; Table 4 and Figure 2B), thus indicating a poorer prognosis in patients with *c-Kit* mutations. Multivariate Cox regression analysis suggested that *c-Kit* mutation could act as an independent prognostic factor (Table 4). Interestingly, we found that all patients with mutations in exon 11 and 13 had an OS of less than 30 weeks, which showed a decreasing tendency in comparison with patients with mutations in other exons, although without statistical significance probably because of the small sample size. All these results indicated that *c-Kit* mutation may act as an adverse prognostic factor for distant metastatic OMMs.

**Table 3:** Clinical characteristics in WT and *c-Kit* mutant patients

Characteristics	Group 1 (n=117)		Group 2 (n=10)		Group 3 (n=12)		*P*		
	No.	%	No.	%	No.	%	1vs2	2vs3	1vs3
Sex									
Male	62	53.0	6	60.0	7	58.3	0.923	1.000	0.724
Female	55	47.0	4	40.0	5	41.6			
Age, years									
Median	57.9±12.6	-	59±12.5	-	57.5±12.6	-	0.798	0.783	0.798
Range	19-75		35-74		28-72				
Primary site									
Palate	70	59.8	7	70.0	8	66.7	0.632	1.000	0.808
Maxillary gum	25	21.4	1	10.0	2	16.7			
Mandible gum	13	11.1	2	20.0	2	16.7			
Other site	9	7.7	0	0.0	0	0.0			
ECOG PS score									
0	39	33.3	4	40.0	4	33.3	0.670	0.821	1.000
1	78	66.7	6	60.0	8	66.7			
Primary lesion									
Positive	18	15.4	2	20.0	3	25.0	1.000	1.000	0.412
Negative	99	84.6	8	80.0	9	75.0			
CLN									
Positive	21	18.0	2	20.0	2	16.7	1.000	1.000	1.000
Negative	96	82.0	8	80.0	10	83.3			
Site of metastases									
Single site	87	74.4	7	70.0	9	75.0	1.000	1.000	1.000
Multiple sites	30	25.7	3	30.0	3	25.0			

**Table 4:** OS of WT and *c-Kit* mutant patients received chemotherapy

Variable	NO.	Median OS (weeks)	*P*	
			Unviariate	Multivariate
Sex				
Male	68	32	0.846	-
Female	59	32		
Age, years				
< 60	56	30	0.845	-
≥ 60	71	33		
Primary site				
Palate	77	32	0.546	-
Maxillary gum	26	34		
Mandible gum	15	30		
Other site	9	35		
ECOG PS score				
0	43	30	0.299	-
1	84	33		
Primary lesion				
Positive	20	26	<0.001	<0.001
Negative	107	34		
CLN				
Positive	23	27	<0.001	<0.001
Negative	104	34		
Site of metastases				
Single site	94	32	0.009	0.192
Multiple sites	33	31		
*c-Kit* mutation				
Positive (Group2)	10	28	0.032	0.013
Negative (Group1)	117	32		

**Figure 3:**
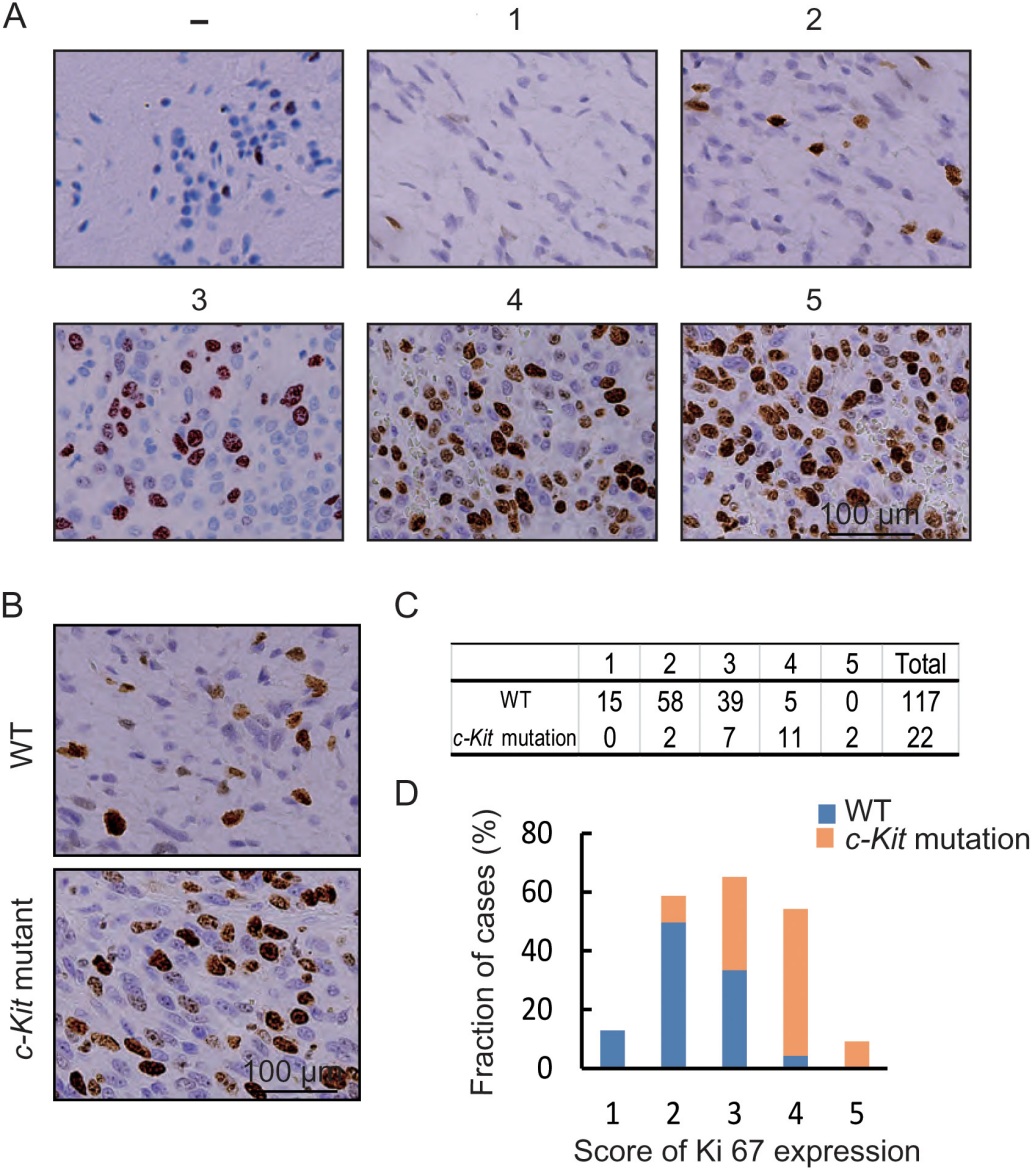
Thecorrelation of *c-Kit* mutations with cell proliferation rate in OMMs **(A)** Representative standard of Ki67 score. **(B)** Immunohistochemical staining of Ki67 in OMMs with WT and mutant *c-Kit* gene. **(C)** Table of Ki67 score in OMMs. **(D)** Bar diagram illustrating the Ki67 score in OMMs with WT (n=117) and mutant *c-Kit* gene (n=22).

The proliferation rate can greatly impact OS in some tumors, and thus the expression of Ki67, the biomarker of proliferation marker, may act as an independent prognostic factor in melanoma [27]. Kit signaling is critical for the proliferation of various cell types including melanoma cells [16, 23, 28]. Thus, we suspected that *c-Kit* mutations could impact the OS of metastatic OMM patients through cell proliferation. As it was impossible to obtain metastatic tumor tissues, we examined cell proliferation rate in the primary melanoma by evaluating the expression of Ki67 using immunohistochemistry (Figure 3A). As shown in Figure 3B-3D, there were more Ki67 positive cells in *c-Kit* mutant patients than in WT patients (*P* < 0.001). These results supported our hypothesis that *c-Kit* mutations could promote cell proliferation in OMMs, thus contributing to the decreased OS.

### Imatinib may benefit metastatic OMM patients with *c-Kit* mutations

**Figure 4:**
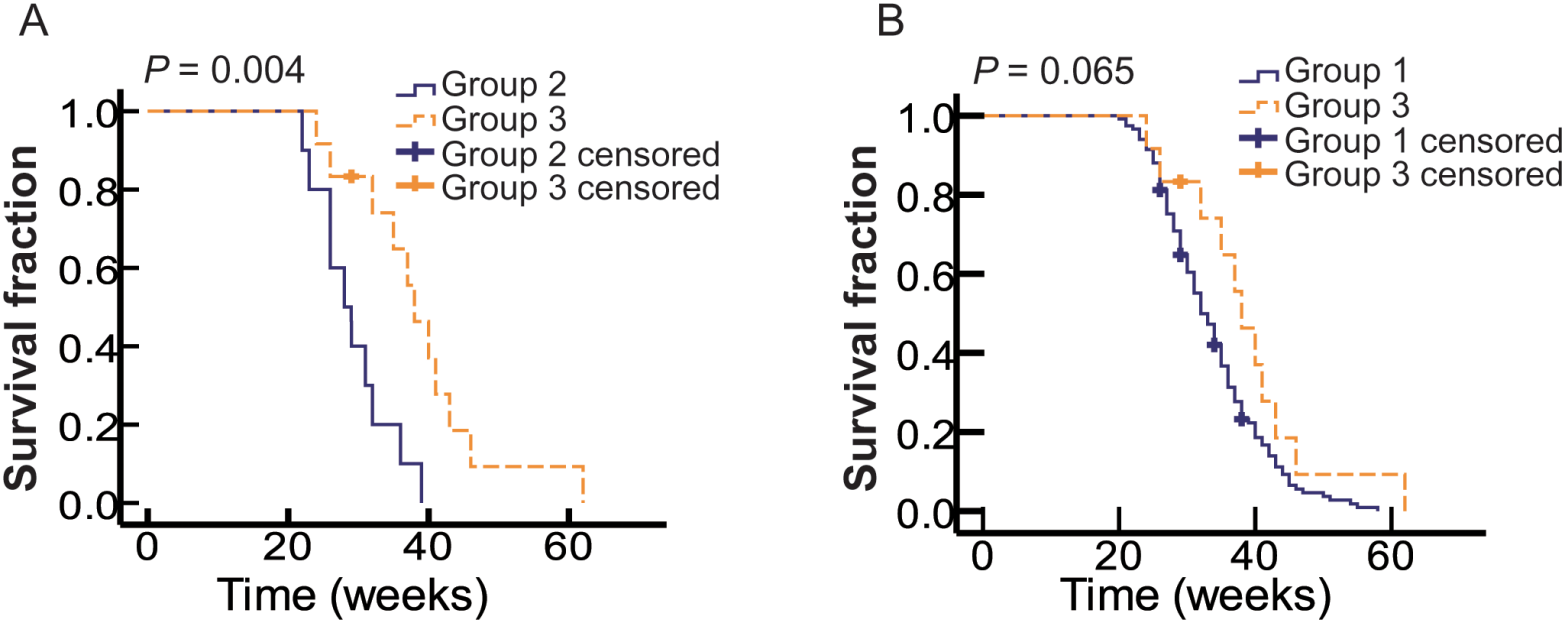
Imatinib increases OS of metastatic OMM patients with *c-Kit* mutations **(A)** Kaplan-Meier curves of OS of patients with *c-Kit* mutations received chemotherapy (Group2, solid blue line, n=10) and imatinib treatment (Group3, dotted yellow line, n=12). **(B)** Kaplan-Meier curves of OS of WT patients (Group1, solid blue line, n=117) and *c-Kit* mutant patients received imatinib treatment (Group3, dotted yellow line, n=12).

To determine whether imatinib was beneficial for metastatic OMM patients with *c-Kit* mutations, we compared the OS of *c-Kit* mutant patients received conventional chemotherapy (Group 2) and imatinib treatment (Group 3). The baseline of these two groups was comparable (Table 3). Both Log-rank test and multivariate Cox regression analysis indicated that patients in Group 3 tended to have better clinical outcomes than patients in Group 2 (Table 5 and Figure 4A). It was interesting that in Group 3, patients with mutations in exon 11 and 13 showed a prolonged, albeit not significant, OS in comparison with patients with other mutations. This could be a true lack of difference or could be attributed to the limited sample size. All these results indicated that imatinib could prolong the OS of metastatic OMM patients with *c-Kit* mutations.

**Table 5:** OS of*c-Kit* mutant patients received chemotherapy and imatinib treatment

Variable	NO.	Median OS (weeks)	*P*	
			Unviariate	Multivariate
Sex				
Male	13	35	0.664	-
Female	9	32		
Age, years				
< 60	10	36	0.351	-
≥ 60	12	29		
Primary site				
Palate	15	32	0.872	-
Maxillary gum	3	32		
Mandible gum	4	36		
ECOG PS score				
0	8	32	0.578	-
1	14	31		
Primary lesion				
Positive	5	24	0.001	0.002
Negative	17	36		
CLN				
Positive	4	24	0.006	0.009
Negative	18	36		
Site of metastases				
Single site	16	35	0.027	0.207
Multiple sites	6	26		
Treatment strategy				
Chemotherapy (Group2)	10	28	0.004	<0.001
Imatinib (Group3)	12	38		

### Drug-resistance reduced the efficacy of imatinib in treating metastatic OMM patients with *c-Kit* mutation

It was interesting that although the media OS of Group 1 was longer than Group 3, there was no significant difference in OS between Group 1 and Group 3 (*P* = 0.065; Figure 4B), which could be attributed to treatment failure of imatinib due to drug resistance in some patients. We found that 5 out of 12 patients in Group 3 suffered from obvious drug resistance, including the enlarging of primary residual lesion, CLNs, distant metastases, and new sites of metastases during the imatinib treatment, and they died shortly after the occurrence of imatinib resistance. Thus, imatinib alone may not be a desirable treatment strategy for OMMs patients with *c-Kit* mutations.

## DISCUSSION

More than 40% of OMM patients died of distant metastasis [8], and it remains a challenge to manage the life-threatening metastasis of OMMs [29, 30]. In this study, we retrospectively analyzed the clinical manifestations, histopathology, treatment strategies, and outcomes of 139 metastatic OMM patients hospitalized in a single institution from Jan. 2008 to Oct. 2015. In line with previous studies on OMMs [8, 30], we also found that the most common site of the primary lesion was the hard palate (61.15%), and that of distant metastasis was the lung (59.3%). The median OS of metastatic OMM patients treated with chemotherapy was only 32 weeks, which was significantly shorter than that of cutaneous melanoma patients [13, 31].

In this study, we found that most metastatic OMM patients survived less than one year, which was shorter than that of cutaneous melanoma patients probably due to the heterogeneity of OMMs [4, 29]. Both residual primary lesion and positive neck nodes may result in a decrease of OS in metastatic OMM patients and could act as independent prognostic factors. OMMs are difficult to completely remove due to the peculiar anatomic structure and function of the stomatognathic system and, as a consequence, residual primary lesion is inevitable in OMMs patients. The residual primary lesion and positive neck nodes may cause a series of fatal complications, including bleeding and difficulty in feeding and breathing, which may result in poor prognosis and even death of these patients.

For many years, systemic chemotherapy has been the primary therapeutic option for patients with metastatic melanoma, but most often the outcomes are disappointing. Some somatic mutations occurring in melanomas, such as mutations in *Braf* and *c-Kit*, are considered as molecular targets for potential therapeutic intervention. Inhibitors of both Braf and c-Kit have been used in the treatment of various cancers, including some subtypes of melanoma [23]. Melanoma is a heterogeneous tumor, and mucosal melanoma differs from cutaneous melanoma in several physical, biological, and genetic characteristics [32]. *Braf* mutations were rare in OMMs, whereas *c-Kit* mutations were frequently detected in OMMs [19, 20]. However, a potential limitation of previous studies was the limited sample size. In this study, we analyzed *c-Kit* mutations in 139 metastatic OMM patients, which had the largest sample size to our knowledge. We found a higher mutation rate (15.8%) than previously reported in Chinese patients [15, 33], probably because all the patients included in our study were metastatic OMM patients. We also found that *c*-*Kit* mutations were mainly located in exon 11 and 13 (63.6%), which was similar to that of mucosal melanoma [20, 26].

In this study, we analyzed the clinical outcomes of metastatic OMMs patients who received conventional chemotherapy. The OS was decreased in *c-Kit* mutant patients in comparison with WT patients, and *c-Kit* mutation may act as a poor prognostic factor of metastatic OMMs. These results were consistent with previous findings [33].

Cell proliferation rate is an important prognostic factor of malignant tumors, and it can be regulated by KIT/MAPK/MEK signaling pathway [34]. We analyzed the cell proliferation rate by evaluating the expression of Ki67 in the primary tumor of metastatic OMM patients, because metastatic tumor tissues of these patients were rarely collected. The Ki67 expression increased significantly in *c-Kit* mutant patients compared with WT patients, indicating an increase in cell proliferation rate. Thus, *c-Kit* mutations may result in poor prognosis in metastatic OMMs through promoting cell proliferation. This, however, needs to be further addressed in future studies.

Imatinib, a c-Kit inhibitor, has been reported to have favorable outcomes in patients with metastatic gastrointestinal stromal tumors. *In vitro* studies have demonstrated the high sensitivity of *c-Kit* mutant cell lines to imatinib [35–37]. However, inconsistent results have also been reported in the treatment of melanomas with imatinib because of the different inclusion criteria of patients [14, 20]. Therefore, whether imatinib is beneficial for metastatic OMM patients remains to be determined. In this study, we found that the OS of metastatic OMM patients received imatinib treatment was prolonged in comparison with patients received chemotherapy, which suggested that imatinib benefited metastatic OMM patients with *c-Kit* mutations, and c-Kit signaling could serve as a molecular therapeutic target in metastatic OMMs.

Although imatinib could act as a prognostic factor and a therapeutic target in metastatic OMMs, there are many factors influencing the effect of imatinib. Patients with *c-Kit* mutations in exon 11 (encodes the juxtamembrane domain) and exon 13 (encodes the tyrosine kinase domain) have been shown to benefit more from the imatinib treatment [26, 38–40]. In this study, we found that patients with *c-Kit* mutations in exon 11 and 13 had a shorter OS compared with patients with other mutations when they were treated with chemotherapy, but a longer OS when they were treated with imatinib. Thus, imatinib had different outcomes in treating different *c-Kit* mutant subgroups. Furthermore, we found that there was no significant difference in OS between WT patients and imatinib-treated *c-Kit* mutations patients, which was consistent with other studies [41, 42]. This less-than-perfect result could be contributed to drug resistance of imatinib, which has been considered as a critical problem in treating patients with *c-Kit* mutations [43]. In our study, 5 out of 12 patients treated with imatinib showed obvious drug-resistant symptoms, which may reduce the efficacy of imatinib treatment. Thus, imatinib resistance should be taken into account and whether the combination of c-Kit inhibitor and chemotherapy could benefit patients with *c-Kit* mutations should be explored in future research.

## PATIENTS AND METHODS

### Patients

This study was approved by the Ethics Committee of Ninth People’s Hospital affiliated to Shanghai Jiaotiong University School of Medicine and all patients were informed for this experimentation. The clinical manifestations, histopathology, treatment, and outcomes of OMM patients with distant metastasis hospitalized in our hospital from Jan. 2008 to Oct. 2015 were retrospectively analyzed. Patients with the following characteristics were eligible for the study: (1) primary OMMs histologically diagnosed by biopsy or surgery specimens by two experienced pathologists; (2) distant metastasis determined by radiological assessment; (3) complete clinical and follow-up records (per 3-6 months); (4) *c-Kit* mutant or wild-type tumors determined by gene screening; and (5) age ≤ 75. Patients (1) with an Eastern Cooperative Oncology Group (ECOG) performance status score ≥ 2 [44]; (2) brain metastasis and/or symptomatic central nervous system metastases; (3) treated by other methods, including radiotherapy; and (4) with other known gene mutations were excluded. The Supplementary Figure 2 showed the process of patients including of the present study. Finally, a total of 139 patients were included in this study.

### Treatment

Upon the diagnosis of OMM, a radical resection of the primary lesion with at least 1.5 cm of healthy tissues was performed in all patients without distant metastasis; and a neck dissection was performed in patients diagnosed with positive CLN by physical exam and confirmed by ultrasound, CT scan, or/and PET CT. Patients were followed-up every 2-4 months for at least 5 years to monitor recurrence or/and metastasis. Once advanced distant metastasis was confirmed, the following treatment would be performed. Before Mar. 2012, for advanced OMM with distant metastasis, chemotherapy with DTIC (dacarbazine injection, Nanjing Pharmaceutical Co. Ltd., Jiangsu, China) and CDDP (cisplatin injection, Qilu Pharmaceutical Co. Ltd., Shandong, China) was repeated every 3 weeks for 4 circles. DTIC was administered on day 2–5 at a dose of 250 mg/m^2^, and CDDP was administered on day 1 at a dose of 75 mg/m^2^ (with hydration). After Mar. 2012, *c-Kit* mutations were screened, and imatinib treatment was recommended to patients with *c-Kit* mutations. For those patients who chose to accept imatinib treatment, imatinib was administered orally at a dose of 400 mg once a day according to previous studies and a glass of water was recommend to minimize gastrointestinal irritation [26]. For those patients without *c-Kit* mutations or who refused imatinib treatment, chemotherapy with DTIC and CDDP described above was applied.

### Histology and immunohistochemistry

The primary disease was diagnosed by biopsy and histology including hematoxylin-eosin staining and immunohistochemical staining of HMB-45, Melan-A, and S-100 protein. Immunohistochemical staining of Ki67, a widely used proliferation marker, was performed in the primary lesion as described previously [27, 45]. Sections were de-waxed and rehydrated, and 3% H_2_O_2_ was used to fade melanin if necessary and block the activity of endogenous peroxidase. Antigen retrieval was performed by heat treating for 15 min. Antibodies against HMB-45, Melan-A, S-100 and Ki67 (1: 200, Santa Cruz Biotechnology, Santa Cruz, CA, USA) were added and incubated overnight at 4°C. The Dako Real Envision Detection System and AEC peroxidase substrate (Dako, Copenhagen, Denmark) were used to detect the primary antibody according to the manufacturer's instructions. To evaluate the nonspecific binding, the primary antibody was substituted with PBS. To evaluate the expression level of Ki67, the following score system was used: 1 for <5% positive cells, 2 for 5-10% positive cells, 3 for 10-30% positive cells, 4 for 30-50% positive cells, and 5 for >50% positive cells, respectively.

### Screening of gene mutations

To select patients suitable for molecular treatment of Kit inhibitor, *c-Kit* mutations were screened in all patients diagnosed as distant metastatic OMMs after Mar. 2012 as described in previous studies [16, 26]. To analyze the effects of *c-Kit* mutations on the prognosis of distant metastatic OMMs, *c-Kit* mutations were screened in distant OMM patients who received chemotherapy from Jan. 2008 to Feb. 2012. To determinate *c-Kit* mutations, genomic DNA was extracted from paraffin-embedded sections with a QIAamp DNA FFPE Tissue Kit (Qiagen, Hilden, Germany). To determinate hotspot mutations, exons 9, 11, 13, 17 and 18 of *c-Kit* gene were amplified by PCR with the genomic DNA. The PCR products were purified by using QIAquick (Qiagen) and directly sequenced with Big Dye Terminator sequencing chemistry on an ABI 3730 automated sequencer (Applied Biosystems, Foster City, CA). All mutations were confirmed by repeating bidirectional sequencing on the ABI sequencer.

### Prognostic variables

The prognostic variables considered in this study included gender, age, primary site, ECOG score, the status and site of distant metastases, the status of CLNs, primary lesions and *c-Kit* mutations. “Primary lesions” indicated the lesions observed in the oral cavity during the treatment and follow-up period. The presence of residual primary lesion in distant metastatic OMMs could be due to patient’s rejection of operation and unresectable lesions. “CLNs” indicated the status of metastasis of CLNs detected by physical exam and then confirmed by ultrasound, CT scan, or/and PET CT upon the diagnosis of distant metastasis. “Site of metastases” referred to the status and number of organ sites involved upon the diagnosis of distant metastasis. The specific organ sites at the time of metastasis were listed in the Supplementary Table 1.

### Statistical analysis

All statistical analyses were performed with SPSS 16.0 for Windows (SPSS Inc., Chicago, IL). Differences between groups were analyzed using Student’s t-test for continuous data, Fisher exact test and chi-square test for categorical data, and Mann-Whitney U test for ranked data, respectively. Survival curves were generated by the Kaplan-Meier method. The prognostic variables included gender, age, primary site, ECOG score, the status and site of distant metastases, CLNs, primary lesions and *c-Kit* mutations. The statistical significance of differences between survival curves was established by the Log-rank test, and multivariate analysis was performed with the Cox proportional hazard model. *P*- values < 0.05 were considered significant, and a 95% confidence interval was used in the Cox regression analysis. OS was defined as the time from the diagnosis of the first distant metastasis to death or the latest follow up.

## CONCLUSIONS

Residual primary lesion, CLNs and *c-Kit* mutations act as adverse prognostic factors of metastatic OMMs. The Kit inhibitor imatinib could benefit metastatic OMM patients with *c-Kit* mutations. We highlight c-Kit as an important target in the research of molecular therapy.

## SUPPLEMENTARY MATERIALS FIGURES AND TABLE



## Abbreviations

OMMs: oral mucosal melanomas
OS: overall survival
CLN: cervical lymph node
ECOG: Eastern Cooperative Oncology Group
