# Supplementary material for: The clinical significance of c-Kit mutations in metastatic oral mucosal melanoma in China
Source: Oncotarget. 2017 Jul 31;8(47):82661–73. doi: 10.18632/oncotarget.19746 (PMC5669918; doi:10.18632/oncotarget.19746)
Supplement: Supplementary file 1 [file oncotarget-08-82661-s001.pdf]

The clinical significance of *c-Kit* mutations in metastatic oral mucosal melanoma in China

SUPPLEMENTARY MATERIALS

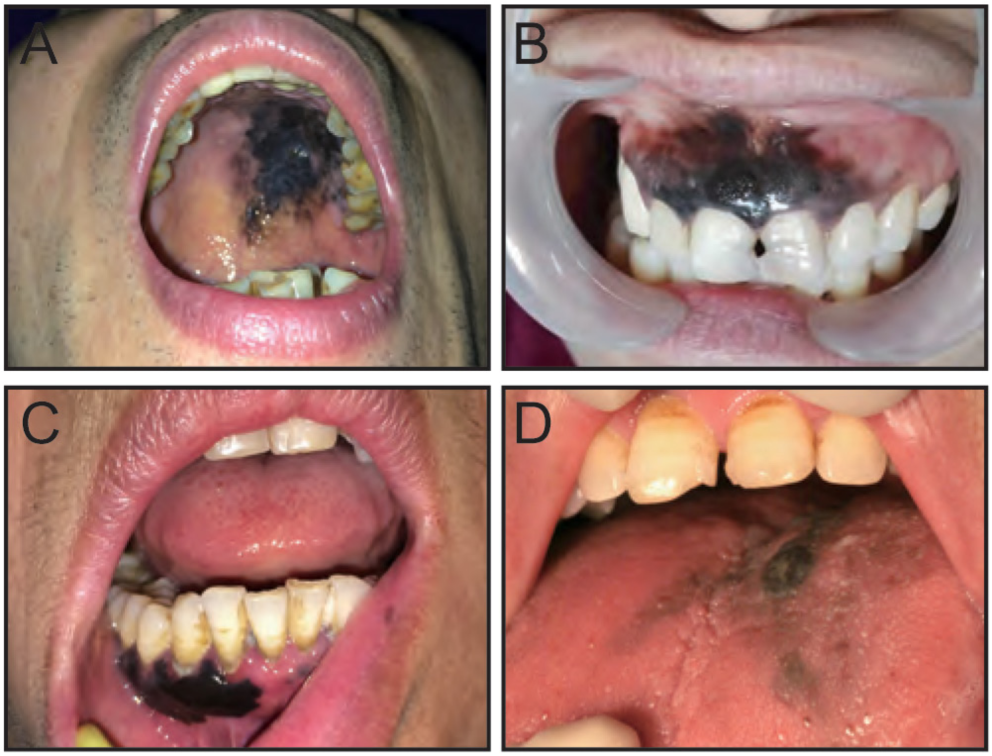

Supplementary Figure 1: Representative views of OMMs in the hard palate **(A),** maxillary gum **(B),** mandible gum **(C)** and tongue **(D).**

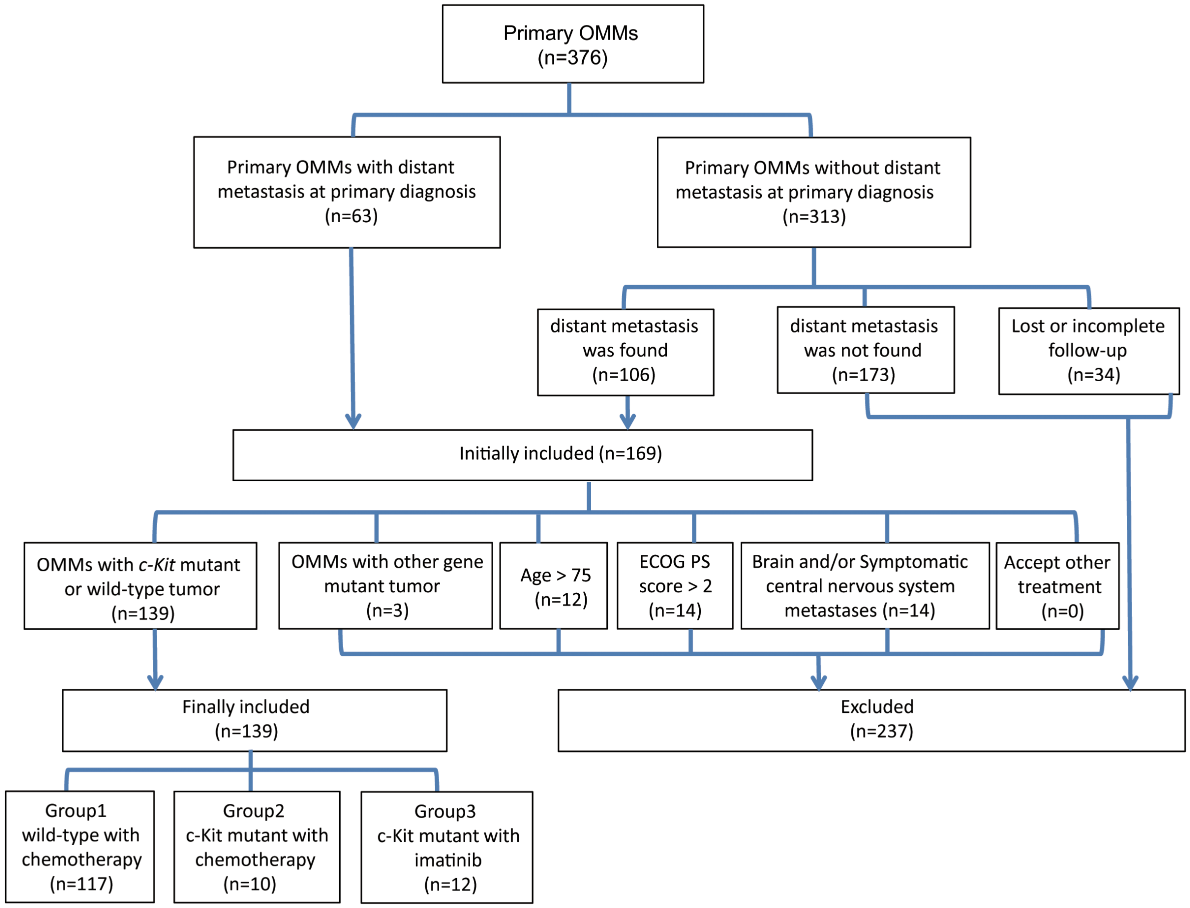

Supplementary Figure 2: Flow diagram of patient selection.

Supplementary Table 1: The specific organ sites at the time of metastasis

Organ of metastases	Group1		Group2		Group3		Total	
	No.	%	No.	%	No.	%	No.	%
Lung	87	58.4	8	61.5	10	66.7	105	59.3
Bone	21	14.1	2	15.4	2	13.3	25	14.1
Liver	29	19.5	3	23.1	3	20.0	35	19.8
Ovary	6	4.0	0	0.0	0	0.0	6	3.4
Other organs	6	4.0	0	0.0	0	0.0	6	3.4
Total organs	149	-	13	-	15	-	177	-
